# Clinical characteristics of hospitalized mild/moderate COVID-19 patients with a prolonged negative conversion time of SARS-CoV-2 nucleic acid detection

**DOI:** 10.1186/s12879-021-05851-z

**Published:** 2021-02-03

**Authors:** Ya Yang, Xiaogang Hu, Lirong Xiong, Peishu Fu, Wei Feng, Wei Li, Liwen Zhang, Fengjun Sun

**Affiliations:** 1 Department of Pharmacy, The First Affiliated Hospital of Army Medical University, Chongqing, 400038 China; 2 Department of Pharmacy, Chongqing University Cancer Hospital, Chongqing, 400030 China; 3 Department of Tuberculosis, Chongqing Public Health Medical Center, Chongqing, 400036 China

**Keywords:** COVID-19, SARS-CoV-2, Clinical characteristics, Negative conversion time

## Abstract

**Background:**

The impact of COVID-19 has been devastating on a global scale. The negative conversion time (NCT) of SARS-CoV-2 RNA is closely related to clinical manifestation and disease progression in COVID-19 patients. Our study aimed to predict factors associated with prolonged NCT of SARS-CoV-2 RNA in mild/moderate COVID-19 patients.

**Methods:**

The clinical features, laboratory data and treatment outcomes of COVID-19 patients were retrospectively analyzed. Then univariate and multivariate analysis were used to screen out risk factors of influencing prolonged NCT of SARS-CoV-2 RNA.

**Results:**

Thirty-two hospitalized mild/moderate COVID-19 patients were enrolled. The general clinical symptoms were cough (78.1%), fever (75%), diarrhea (68.8%), expectoration (56.3%), and nausea (37.5%). More than 40% of the patients had decreased erythrocyte, hemoglobin and leucocyte and 93.8% patients were detected in abnormalities of chest CT. The median NCT of SARS-CoV-2 RNA was 19.5 days (IQR: 14.25–25). Univariate analysis found fever, nausea, diarrhea and abnormalities in chest CTs were positively associated with prolonged NCT of viral RNA (*P*< 0.05). The multivariate Cox proportional hazard model revealed that fever [Exp (B), 0.284; 95% CI, 0.114–0.707; *P*<0.05] and nausea [Exp (B), 0.257; 95%CI, 0.096–0.689; *P*<0.05] were two significant independent factors.

**Conclusions:**

Fever and nausea were two significant independent factors in prolonged NCT of viral RNA in mild/moderate COVID-19 patients, which provided a useful references for disease progression and treatment of COVID-19.

**Supplementary Information:**

The online version contains supplementary material available at 10.1186/s12879-021-05851-z.

## Background

In December 2019, novel pneumonia of unknown causes was reported in Wuhan, Hubei province in China. On February 11, 2020, the World Health Organization (WHO) officially named the disease as Coronavirus Disease 2019 (COVID-19), which caused by Severe Acute Respiratory Syndrome Coronavirus-2 (SARS-CoV-2). Following the initial outbreak, COVID-19 has become a global public health emergency of international concern [1]. There had been 23 million confirmed cases, and 800,000 confirmed deaths by 23 August 2020 spreaded across 216 countries and territories [2]. The number of COVID-19 patients has increased exponentially, causing devastation on a global scale [3].

According to the severity of the disease, COVID-19 patients were divided into asymptomatic, mild, moderate and severe cases. Mild/moderate COVID-19 patients has mild clinical symptoms and will develop into severe cases without timely treatment [4]. Therefor, it is very important to control the disease progression of mild/moderate patients. The standard of cure is the relief of symptoms and two successive negative viral nucleic acid [4]. The negative conversion of SARS-CoV-2 RNA was essential in the discharge criteria during hospitalization [5, 6]. The negative conversion time (NCT) of SARS-CoV-2 RNA is closely related to clinical manifestation and disease progression in COVID-19 patients. Therefore, identification of factors associated with the NCT in COVID-19 could contribute to the disease progression and clinical outcomes. Hu et al. reported that older age and chest tightness were independently associated with delayed clearance of SARS-CoV-2 RNA in hospitalized patients [7]. The COVID-19 patients with digestive symptoms are more likely to test positive for viral RNA in a stool sample and have a longer delay before viral clearance than patients with only respiratory symptoms [8]. Several studies also focused on the effects of impaired immunity and blood system on NCT in COVID-19 patients. Wang et al. reported neutrophil to CD4+ lymphocyte ratio is a potential and useful biomarker for predicting the NCT in COVID-19 patients [9]. The integrated indicator of leucocytes, neutrophils and CD3+CD4+ lymphocytes showed a good performance in predicting the negative conversion [10]. Preliminary clinical treatment showed that some drugs, such as chloroquine, effectively potentiated virus clearance in COVID-19 patients [11]. However, there is limited data regarding the potential predictors of NCT in mild/moderate COVID-19 patients.

Therefore, this study analyzed clinical characteristics of NCT of SARS-CoV-2 RNA in mild/moderate COVID-19 patients, We hoped to find out the risk factors affecting NCT of SARS-CoV-2 RNA, which provided a useful references for disease progression and treatment of COVID-19.

## Methods

### Subjects and data collection

The clinical data and laboratory test results were analyzed retrospectively from 32 mild/moderate COVID-19 patients admitted to the Public Health Medical Center and Prevention of Chongqing from January 31 to February 28, 2020. All patients were identified to be nucleic acid positive for SARS-CoV-2 and were convalescent before hospital discharge.

Data was collected and recorded from each patient, which included demographic and clinical information (gender, age, smoking history, and comorbidities), clinical manifestations (fever, coughing, expectoration, anhelation, myalgia, nausea and vomiting, diarrhea, osphyalgia, etc.), and laboratory test results (standard blood counts, procalcitonin, C-reactive protein, blood biochemistry, coagulation function, and myocardial enzyme spectrum, etc). The normal range of laboratory examination is the standard of Reference Range Values for National Practice for Clinical Testing (4th Edition) published by People’s Medical Publishing House [12]. Chest CT and therapeutic drug use were also documented. Then, the factors associated with NCT of SARS-CoV-2 RNA in upper respiratory specimens were analyzed.

### Therapeutic strategies

Strategies of treatment for COVID-19 patients were shown in supplementary Figure 1. The primary treatment strategy is combined antiviral therapy, which mainly comprises interferon-α2b(IFN-α2b)and lopinavir/ritonavir (LPV/r) (group 1), IFN-α2b, LPV/r and ribavirin (group 2), IFN-α2b and ribavirin (group 3). When SARS-CoV-2 RNA was still detectable after 19 days of drug therapy, we discontinued current antiviral drugs and switched to combination of IFN-α2b and chloroquine phosphate which was defined as group 4(combination of IFN-α2b and Ribavirin, then combination of IFN-α2b and chloroquine phosphate) and group 5 (IFN-α2b and LPV/r, then combination of IFN-α2b and chloroquine phosphate) until negative conversion of SARS-CoV-2 RNA.

### Definitions of basic concepts

Throat swab specimens were collected from each patient every other day during hospitalization. RT-PCR assay was used to confirm COVID-19 patients through detecting the RNA of SARS-CoV-2 in throat swab samples. Patients with consecutive positive nucleic acid tests were confirmed as SARS-CoV-2 infection. The standard of negative conversion is two successive negative viral nucleic acid detection tests in 24 h minimum sampling intervals. The term convalescent patients refer to recovered afebrile patients without respiratory symptoms who had two successive (minimum 24 h sampling interval) negative results for SARS-CoV-2 RNA from oropharyngeal swabs by RT-PCR.

### Statistical analysis

Normally distributed continuous variables are summarized as the mean mean (standard deviation, SD), otherwise, median (interquartile range, IQR) was used to describe. Categorical variables are expressed using numbers and percentages. Cox regression analysis was used to analyze the factors. First, univariate analysis was performed, and the indicators with statistical significance were analyzed for Kaplan-Meier survival analysis. The Log Rank method was used to compare the differences between groups. A Cox proportional hazard model was used for multivariate analysis. IBM SPSS Statistics 26 was used for statistical analysis and survival image rendering. *P*< 0.05 was considered to indicate a statistically significant difference.

## Results

### The relationship between demographic and clinical information and NCT of SARS-CoV-2 RNA

Of these patients, males accounted for 43.8% (14) of confirmed cases, and 56.3% (18) were females. The median age of males and females was 34.00 (29.00, 47.00) years and 43.00 (37.75, 57.25) years respectively, suggesting that middle-aged people were more susceptible to infection than other age groups. A total of 12.5% of patients were smokers. More than a quarter of patients (34%) had underlying diseases, including cardiovascular and cerebrovascular diseases (25%), gastrointestinal disease (15.6%), respiratory disease (12.5%) and a neurological disorder (3.1%). All patient data were analyzed using the Cox regression analysis for univariate analysis. However, we found that no demographic or clinical information had a significant positive association with NCT of viral RNA in patients (*P*> 0.05, Table 1).

**Table 1:** Univariate analysis of demographic and clinical information on NCT of SARS-CoV-2 RNA in 32 COVID-19 patients

Factors	Count n (%)	*p* value	Exp(B)	95.0% Cl for Exp(B)	
				lower limit	detection limits
**Demographic and clinical information**					
Age breakdown					
<45	21 (65.6)	0.636	1.190	0.579	2.446
>45	11 (34.4)				
Sex					
Male	14 (43.8)	0.689	1.325	0.398	4.411
Female	18 (56.3)				
Smoking	4 (12.5)	0.988	0.992	0.343	2.868
Underlying diseases					
Hypertension	3 (9.4)	0.710	1.257	0.376	4.197
Diabetes	1 (3.1)	0.110	5.750	0.671	49.244
Coronary heart disease	1 (3.1)	0.252	3.373	0.421	26.993
Viral hepatitis type B	2 (6.3)	0.399	0.536	0.126	2.282
Hyperlipemia	2 (6.3)	0.199	0.550	0.221	1.370
Cerebral hemorrhage	1 (3.1)	0.593	0.578	0.078	4.305
Chronic bronchitis	1 (3.1)	0.110	5.750	0.671	49.244
Depression	1 (3.1)	0.075	7.340	0.820	65.691
Pneumonia	1 (3.1)	0.258	0.312	0.042	2.342
**Clinical manifestation**					
The duration from disease onset to hospital	32 (100)	0.345	1.057	0.940	1.190
Fever	24 (75.0)	0.050*	0.431	0.186	1.000
Cough	25 (78.1)	0.564	0.779	0.334	1.818
Expectoration	18 (56.3)	0.335	0.707	0.349	1.431
Anhelation	11 (34.4)	0.714	0.871	0.416	1.822
Throat discomfort	11 (34.4)	0.669	0.852	0.409	1.775
Headache and dizziness	7 (21.9)	0.323	0.650	0.277	1.526
Myodynia	7 (21.9)	0.123	0.491	0.199	1.213
Osphyalgia	2 (6.3)	0.341	0.495	0.117	2.103
Diarrhea	22 (68.8)	0.007*	0.318	0.138	0.733
Ventosity	2 (6.3)	0.524	0.622	0.145	2.677
Nausea	12 (37.5)	0.002*	0.262	0.112	0.613
Vomiting	3 (9.4)	0.314	2.161	0.482	9.686
Muscular stiffness	5 (15.6)	0.235	0.559	0.214	1.460
Chills	2 (6.3)	0.126	3.288	0.716	15.105
Fatigue	9 (28.1)	0.509	0.768	0.350	1.683
Snot	3 (9.4)	0.123	0.387	0.113	1.329
Insomnia	5 (15.6)	0.444	0.678	0.250	1.836
Chest tightness	5 (15.6)	0.978	1.014	0.385	2.670

Data are shown as n (%) or IQR unless specified otherwise

The Log Rank method was used to compare the differences between groups, *p* < 0.05 was considered to indicate a statistically significant difference (indicated by *)

### The relationship between clinical manifestation and NCT of SARS-CoV-2 RNA

The median duration from disease onset to hospital admission was 3.5 (IQR: 2–6) days, with a median of 20 days from illness onset to hospital discharge (IQR: 15.25–26) and the median time from positive to negative conversion of SARS-CoV-2 RNA was 19.5 days (IQR: 14.25–25). The majority of the patients showed an initial symptom of fever (75%), but a quarter of the patients were afebrile, alerting the need for the caution of atypical cases. In addition to fever, other clinical symptoms mainly included fatigue (78.1%), expectoration (56.3%), diarrhea (68.8%), nausea (37.5%), anhelation (34.4%), and throat discomfort (34.4%). Clinical manifestations of the study population are summarized in Table 1.

**Fig. 1:**
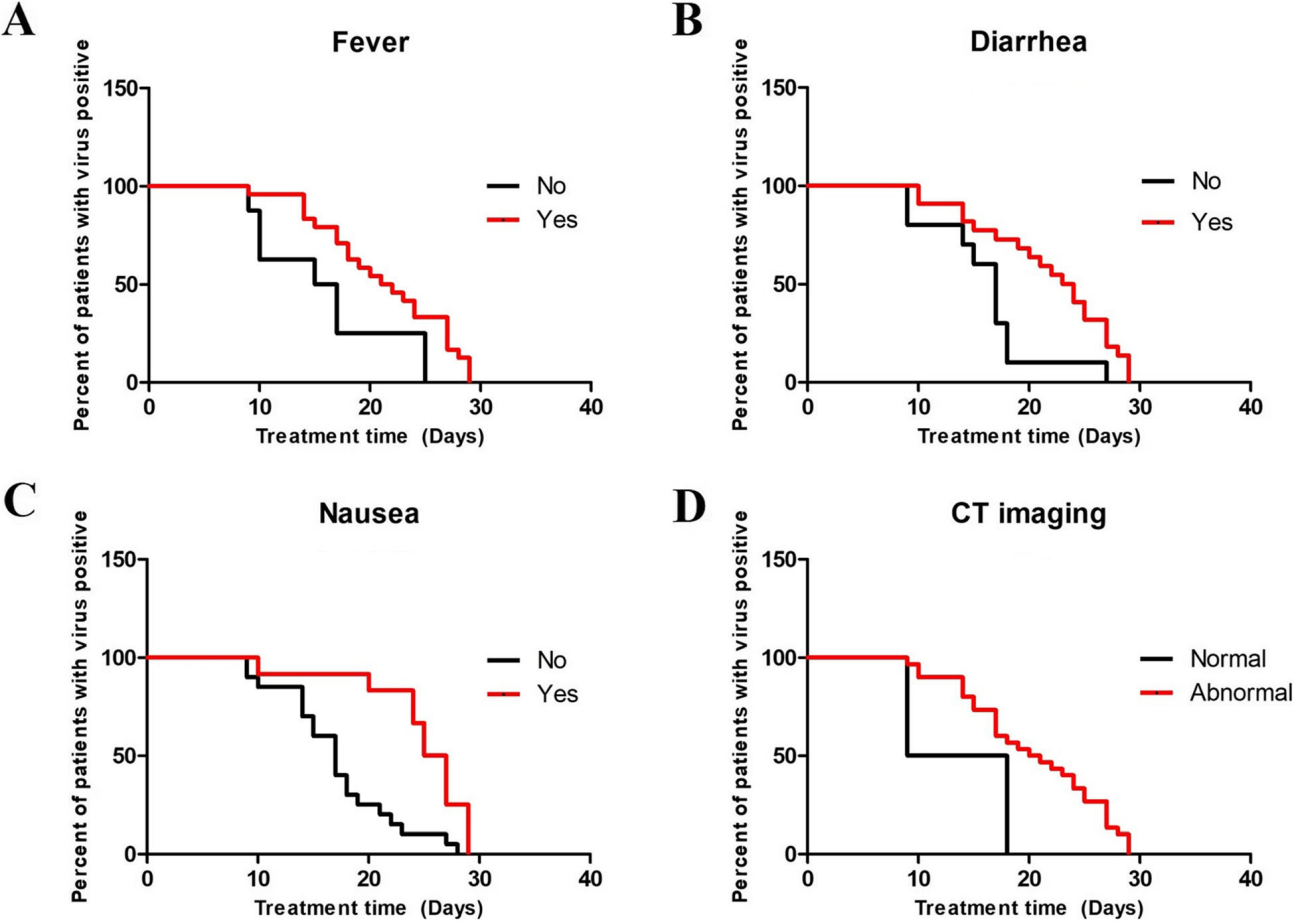
Kaplan-Meier curves for NCT of SARS-CoV-2 RNA according to clinical characteristics of COVID-19 patients. **a** Fever was positively associated with NCT of viral RNA in patients [Exp (B), 0.431; 95% CI, 0.186–1.000; *P*=0.035]; **b** Diarrhea was positively associated with NCT of viral RNA in patients [Exp (B), 0.262; 95% CI, 0.112–0.613; *P*=0.002]; **c** Diarrhea was positively associated with NCT of viral RNA in patients [Exp (B), 0.318; 95% CI, 0.138–0.733; *P*=0.007]; **d** Abnormalities in chest CTs were positively associated with NCT of viral RNA in patients [Exp (B), 0.209; 95% CI, 0.046–0.957; *P*=0.044]

The Cox regression analysis was used for univariate analysis; we found that fever, nausea, and diarrhea were a significant positive association with NCT of viral RNA in patients (*P*< 0.05, Table 1). The Kaplan-Meier curves revealed that fever, nausea, and diarrhea had a significantly prolonged NCT of SARS-CoV-2 RNA compared with the normality’s group (*P*< 0.05; Fig. 1a, b, c).

### The relationship between laboratory findings and NCT of SARS-CoV-2 RNA

The laboratory inspection section showed that 43.8% of the patients developed leucopenia, and 28.1% of patients had lymphocytopenia. The hemoglobin, platelets, and albumin levels were lower in 53.1, 37.5, and 34.4% of patients, respectively. The levels of procalcitonin, C-reactive protein, total bilirubin, direct bilirubin, creatinine, lactic dehydrogenase were increased in 9.4, 40.6, 40.6, 46.9, 25, and 21.9% of patienst, respectively. All patient data were analyzed using the Cox regression analysis for univariate analysis. However, there were no significant findings with a positive association with NCT of viral RNA in patients (*P*> 0.05, Supplementary Table 1).

### The relationship between radiological findings and NCT of SARS-CoV-2 RNA

Ground-glass opacity (GGO) and high-density shadow were the typical radiological findings on chest CT scan in COVID-19 patients. Abnormalities in chest CT were detected in 93.8% of patients. A total of 87.5% of patients had ground-grass opacities over bilateral lungs, which was the most common pattern of CT changes and corresponded to pathological diffuse alveolar damage [13].

We analyzed the univariate analysis using the Cox regression and found that abnormalities in chest CT were a significant positive association with NCT of viral RNA in patients (*P*< 0.005, Table 2). The Kaplan Meier curves revealed that abnormalities in chest CT had a significantly prolonged NCT of SARS-CoV-2 RNA compared with normality’s group (*P*< 0.05; Fig. 1d).

**Table 2:** The relationship between radiological findings^a^ and NCT of SARS-CoV-2 RNA in 32 patients with COVID-19

Factors	Count n (%)	*p* value	Exp(B)	95.0% Cl for Exp(B)	
				lower limit	detection limits
Total	32 (100)				
Abnormal	30 (93.8)	0.044*	0.209	0.046	0.957
Involved lung field					
Unilateral	8 (25)	0.202	0.570	0.241	1.350
Bilateral	22 (68.8)	0.503	1.314	0.591	2.920
Radiological characteristics					
Ground-grass opacities	28 (87.5)	0.940	0.960	0.331	2782
High-density shadow	17 (53.1)	0.083	0.536	0.265	1.086

Data are shown as n (%) unless specified otherwise

The Log Rank method was used to compare the differences between groups, *p* < 0.05 was considered to indicate a statistically significant difference (indicated by *)

### Taking antiviral medications during hospitalization

The treatment strategy and the NCT were shown in supplementary Figure 1. The median time from positive to negative conversion of SARS-CoV-2 RNA was 19.5 days (IQR: 14.25–25). The univariate analysis using the Cox regression and found that combined treatment of IFN-α2b and LPV/r (group 1) had a significantly shorten NCT of SARS-CoV-2 RNA ([Exp (B),12.522; 95% CI, 3.389 46.263; *P*<0.05]), which is consistent with other clinical treatments [14]. But combination of IFN-α2b and chloroquine phosphate (group 5) had a significantly prolonged NCT of SARS-CoV-2 RNA ([Exp (B),0.388; 95% CI, 0.182 0.827; *P*<0.05]), which group had a high percentage of gastrointestinal symptoms such as nausea and diarrhea (Data not shown) that may be risk factors for NCT extension. Treatment of groups 2, 3 and 4 didn’t shorten or prolong the NCT (*P*> 0.05).

### Multivariate analysis of risk factors of influencing prolonged NCT of SARS-CoV-2 RNA

We had summarized the results of the univariate analysis that fever, nausea, diarrhea, and abnormalities in chest CT had a significantly prolonged NCT of SARS-CoV-2 RNA. To analyze factors on NCT of SARS-CoV-2 RNA systematically, the multivariate Cox’s proportional hazard model was used to analyze the multivariate analyses. We revealed that fever [Exp (B), 0.284; 95% CI, 0.114–0.707; *P*< 0.005] and nausea [Exp (B), 0.257; 95%CI, 0.096–0.689; *P*< 0.005] were independent risk factors of prolonged NCT of SARS-CoV-2 RNA in patients with COVID-19 (Table 3).

**Table 3:** Independent factors of NCT of SARS-CoV-2 RNA by the multivariate Cox’s proportional hazard model

Factors	*p* value	Exp(B)	95.0% CI for Exp(B)	
			lower limit	detection limits
Fever	0.007*	0.284	0.114	0.707
Nausea	0.007*	0.257	0.096	0.689
Diarrhea	0.376	0.648	0.248	1.692
CT imaging	0.150	0.299	0.058	1.550

A Cox proportional hazard model was used for multivariate analysis, *p* < 0.05 was considered to indicate a statistically significant difference (indicated by *)

## Discussion

COVID-19 is a global pandemic that has rapidly spread worldwide since reported in December 2019. A negative nucleic acid test of SARS-CoV-2 is the standard for a cure of COVID-19. To investigate the timeline of RNA negative conversion of COVID-19 patients, this study retrospectively analyzed clinical characteristics in a cohort of mild/moderate hospitalized patients in Chongqing, China. We found that fever, nausea, diarrhea, and abnormalities in chest CT were correlated to a prolonged NCT of SARS-CoV-2 RNA. More importantly, fever and nausea were the independent factors predicting NCT of mild/moderate COVID-19 patients.

Fever, a respiratory manifestation, is the most commonly reported symptom in patients infected with SARS-CoV-2 [15, 16]. Accordingly, fever may be a clinical sign of poor prognosis in patients and response to the release of inflammatory mediators such as cytokines and chemokines [17, 18]. These inflammatory mediators cause tissue damage and organ dysfunction by stimulating toxic oxygen derivatives suggesting that the NCT of SARS-CoV-2 RNA may be prolonged in fever patients [19, 20].

Chest CT has a high sensitivity to detect lung abnormalities, which is quite helpful in the early diagnosis of the disease. The “Diagnosis and Treatment Scheme for Coronavirus Disease (Trial Version 7)” recommended that a CT examination serves as the diagnostic basis for COVID-19. For chest imaging in patients with COVID-19, the early manifestation was multiple plaque shadows and interstitial changes. The later development was multiple ground glass shadows and infiltration shadows in both lungs. In severe cases, lung consolidation can occur, presenting as “white lung,” which may be related to the immunopathology. Most studies have suggested that a dysregulated/exuberant innate responses are the primary cause of coronavirus-mediated pathology [21]. Many cytokines or chemokines are involved in the immune storm after the infection of coronavirus, which eventually leads to lung injury and acute respiratory distress syndrome [22]. The improvement of chest CT was after that of body temperature. It preceded the negative conversion of nucleic acid tests, suggesting that abnormalities in chest CT could indirectly reflect the persistence of SARS-CoV-2 RNA in patients with COVID-19. Our study found that abnormalities in chest CT could prolong the NCT of patients with COVID-19. Therefore, the improvement of chest CT as soon as possible has an essential effect on shortening NCT in patients with COVID-19 and promoting patients to be discharged more rapidly.

Although COVID-19 most commonly presents with respiratory symptoms, such as cough and shortness of breath [15, 23, 24], there is evidence that the illness can also present with nonrespiratory symptoms, most notably digestive symptoms such as diarrhea, diminished appetite, nausea, and vomiting [25–27]. Diarrhea appeared to be the most common GI complaint [28], followed by nausea and vomiting [23]. SARS-CoV-2 RNA was detected in stool samples from COVID-19 patients for the first reported in the United States [29]. Viral RNA was still positive in gastrointestinal specimens even after virus RNA levels could not be detected in respiratory samples [28], implying direct infectivity of the virus on the intestinal tract. Current research shows that the primary target organ of COVID-19 is the lung, but clinical evidence suggests that the gastrointestinal tract may be another viral target organ.

The SARS-CoV-2 receptor angiotensin-converting enzyme 2 (ACE2) has been found in both upper and lower gastrointestinal tract where its expression level was nearly 100 times higher than that of respiratory organs [30, 31]. Patients with symptoms of the digestive system have more viruses in their gut [8], and maybe more likely to cause direct damage to the intestinal mucosa. Our research found that digestive symptoms, including nausea and diarrhea, are the factors of NCT of COVID-19 patients, suggesting that patients with gastrointestinal symptoms should seek medical care to avoid delayed diagnosis and prolong treatment time.

There are several limitations to our study. First, the sample size of this study is not large enough. Second, we have not conducted randomized controlled trials, so we cannot determine the therapeutic effect of chloroquine on COVID-19 and its impact on shortening the time of negative viral conversion.

## Conclusion

In conclusion, our study suggests that fever, nausea, diarrhea, abnormalities in chest CT and are risk factors for prolonged NCT of SARS-COV-2 RNA in mild/moderate COVID-19 patients. More importantly, fever and nausea are the independent factors in predicting prolonged NCT of SARS-CoV-2 RNA. Meanwhile, we should pay attention to gastrointestinal symptoms and provide timely treatment. This study provide a useful references for disease progression and treatment of COVID-19.

## Supplementary Information

**Additional file 1: Supplementary Figure 1.** Schematic illustration of the treatment strategy and days medication use during hospitalization in 32 COVID-19 patients. Group 1: patient number 1–11. Combined treatment of IFN-α2b and LPV/r. Group 2: patient number 12–17. Combined treatment of IFN-α2b, LPV/r and ribavirin, Group 3: patient number 18–19. Combined treatment of IFN-α2b and ribavirin. Group 4: patient number 20–21. Combined treatment of IFN-α2b and ribavirin for 19 days and switched to combination of IFN-α2b and chloroquine phosphate. Group 5: patient number 22–32. Combined treatment of IFN-α2b and LPV/r for 19 days and switched to combination of IFN-α2b and chloroquine phosphate.

**Additional file 2: Supplementary Table 1.** The relationship between laboratory findings and NCT of SARS-CoV-2 RNA in 32 patients with COVID-19

## Data Availability

The datasets used and/or analyzed during the current study are available from the corresponding author on reasonable request.

## Abbreviations

COVID-19: Coronavirus disease 2019
SARS-CoV-2: Severe Acute Respiratory Syndrome Coronavirus-2
NCT: Negative conversion time
RT-PCR: Reverse Transcription-polymerase Chain Reaction
CT: Computerized tomography
IFN-α2b: Interferon-α2b
IQR: Interquartile range
GGO: Ground-glass opacities
