# Supplementary figures and images for: Clinical characteristics of hospitalized mild/moderate COVID-19 patients with a prolonged negative conversion time of SARS-CoV-2 nucleic acid detection
Source: BMC Infect Dis. 2021 Feb 3;21:141. doi: 10.1186/s12879-021-05851-z (PMC7856599; doi:10.1186/s12879-021-05851-z)

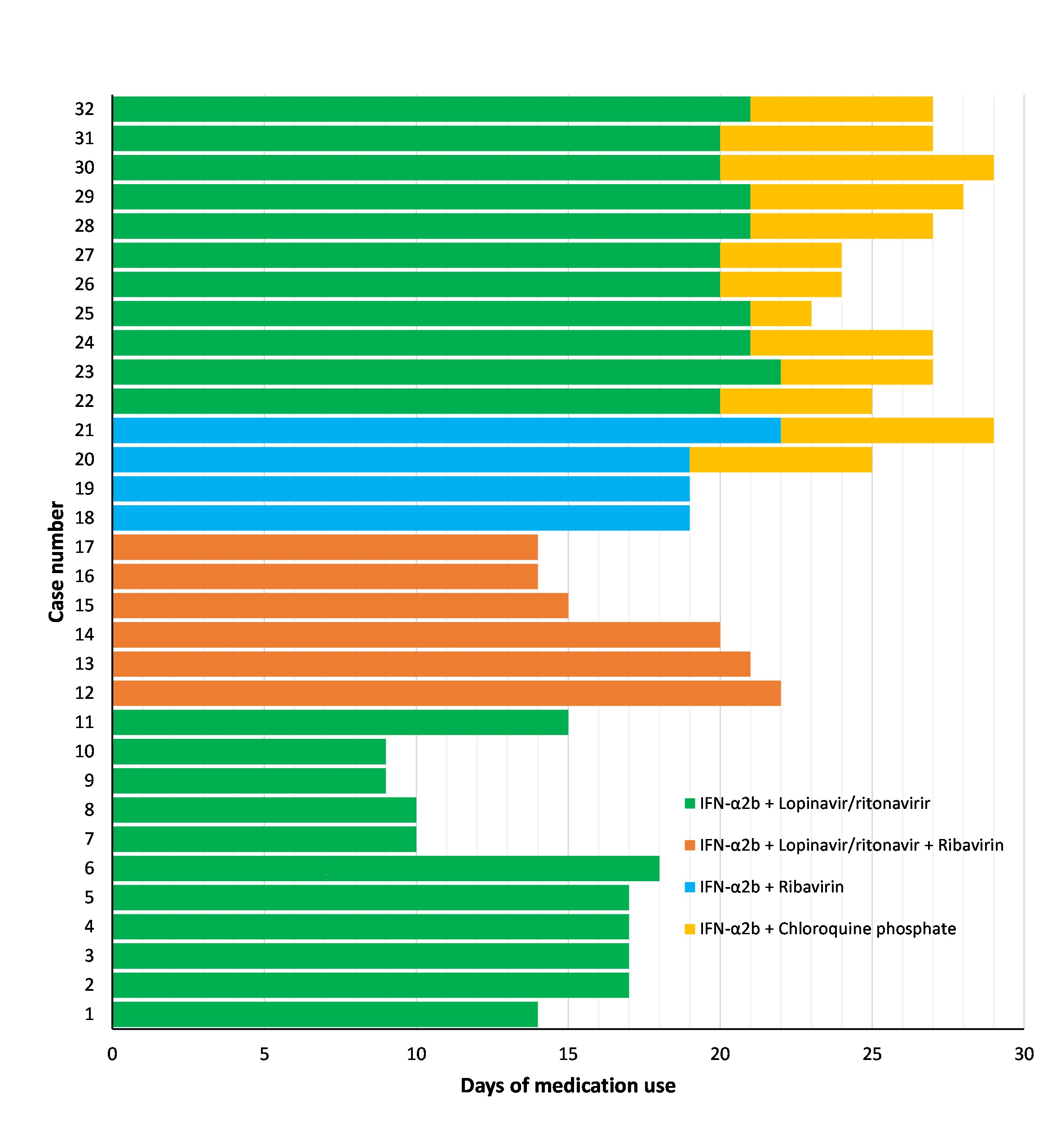

Supplement: Supplementary file 1 — Additional file 1: Supplementary Figure 1. Schematic illustration of the treatment strategy and days medication use during hospitalization in 32 COVID-19 patients. Group 1: patient number 1–11. Combined treatment of IFN-α2b and LPV/r. Group 2: patient number 12–17. Combined treatment of IFN-α2b, LPV/r and ribavirin, Group 3: patient number 18–19. Combined treatment of IFN-α2b and ribavirin. Group 4: patient number 20–21. Combined treatment of IFN-α2b and ribavirin for 19 days and switched to combination of IFN-α2b and chloroquine phosphate. Group 5: patient number 22–32. Combined treatment of IFN-α2b and LPV/r for 19 days and switched to combination of IFN-α2b and chloroquine phosphate. [file 12879_2021_5851_MOESM1_ESM.jpg]
