# Supplementary material for: Clinical characteristics of hospitalized mild/moderate COVID-19 patients with a prolonged negative conversion time of SARS-CoV-2 nucleic acid detection
Source: BMC Infect Dis. 2021 Feb 3;21:141. doi: 10.1186/s12879-021-05851-z (PMC7856599; doi:10.1186/s12879-021-05851-z)
Supplement: Supplementary file 2 — Additional file 2: Supplementary Table 1. The relationship between laboratory findings and NCT of SARS-CoV-2 RNA in 32 patients with COVID-19 [file 12879_2021_5851_MOESM2_ESM.docx]

Supplementary table 1. The relationship between laboratory findings and NCT of SARS-CoV-2 RNA in 32 patients with COVID-19

| Factors | Count  n (%) | p value | Exp(B) | 95.0% Cl for Exp(B) | |
| --- | --- | --- | --- | --- | --- |
|  |  |  |  | lower limit | detection limits |
| Total | 32(100) |  |  |  |  |
| Leukocyte count decrease (↓) | 14(43.8) | 0.176 | 0.612 | 0.301 | 1.246 |
| Neutrophil count decrease (↓) | 8(25) | 0.661 | 0.836 | 0.375 | 1.864 |
| Neutrophil percentage decrease (↓) | 9(28.1) | 0.171 | 0.581 | 0.267 | 1.263 |
| Lymphocyte count decrease (↓) | 12(37.5) | 0.959 | 1.019 | 0.494 | 2.102 |
| Lymphocyte percentage decrease (↓) | 9(28.1) | 0.529 | 1.275 | 0.598 | 2.718 |
| Erythrocyte count decrease (↓) | 24(75) | 0.942 | 1.031 | 0.453 | 2.349 |
| Hemoglobin decrease (↓) | 17(53.1) | 0.678 | 0.858 | 0.417 | 1.765 |
| packcd cell  volume (↓) | 20(62.5) | 0.722 | 1.140 | 0.554 | 2.344 |
| Platelet count decrease (↓) | 12(37.5) | 0.140 | 1.823 | 0.820 | 4.052 |
| Procalcitonin increase (↑) | 3(9.4) | 0.536 | 1.477 | 0.429 | 5.091 |
| C-Reactive Protein increase (↑) | 13(40.6) | 0.492 | 1.302 | 0.614 | 2.761 |
| Total Protein (↓) | 21(65.6) | 0.934 | 1.032 | 0.494 | 2.155 |
| Albumin decrease (↓) | 11(34.4) | 0.824 | 0.920 | 0.442 | 1.917 |
| Total bilirubin increase (↑) | 13(40.6) | 0.297 | 1.465 | 0.715 | 3.000 |
| Direct bilirubin increase (↑) | 15(46.9) | 0.339 | 1.411 | 0.697 | 2.859 |
| Creatinine increase (↑) | 8(25) | 0.936 | 0.968 | 0.433 | 2.163 |
| Lactic dehydrogenase increase (↑) | 7(21.9) | 0.993 | 1.004 | 0.433 | 2.327 |
| D-Dimer increase (↑) | 2(6.3) | 0.783 | 1.227 | 0.286 | 5.260 |

Data are shown as n (%) unless specified otherwise.

The Log Rank method was used to compare the differences between groups, p < 0.05 was considered to indicate a statistically significant difference (indicated by *).
